# Supplementary material for: Splice-Junction-Based Mapping of Alternative Isoforms in the Human Proteome
Source: Cell Rep. Author manuscript; Available in PMC 2020 Jan 15. (PMC6961840; doi:10.1016/j.celrep.2019.11.026)
Supplement: 2 [file NIHMS1546469-supplement-2.zip › DF1/PXD010154/Lung/Lung_22_MYH11_QCQGQAAQEAAGGGR.pdf]

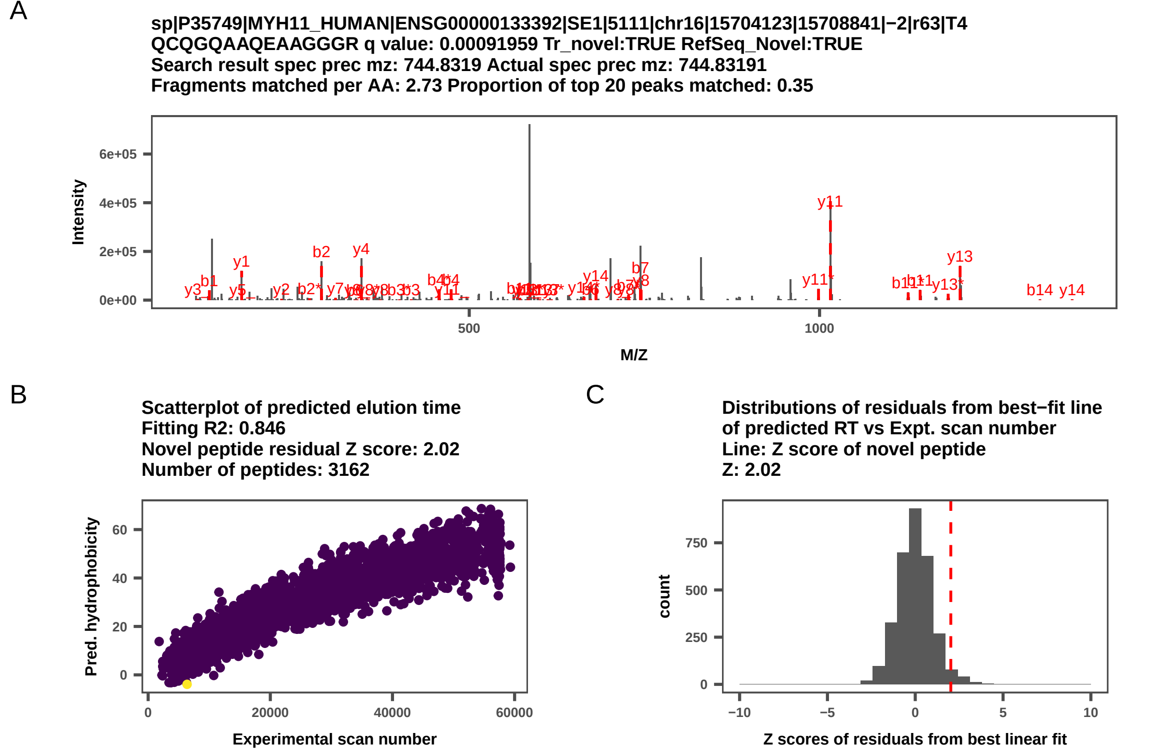
A
sp|P35749|MYH11_HUMAN|ENSG00000133392|SE1|5111|chr16|15704123|15708841|−2|r63|T4
QCQGQAAQEAAGGGR q value: 0.00091959 Tr_novel:TRUE RefSeq_Novel:TRUE
Search result spec prec mz: 744.8319 Actual spec prec mz: 744.83191
Fragments matched per AA: 2.73 Proportion of top 20 peaks matched: 0.35
Intensity
6e+05
4e+05
2e+05
0e+00
M/Z
500
1000
y11
y13
b2
y4
y1
b1
y3
y5
y2
b7
y14
y11*
y13*
b14
y14
B
Scatterplot of predicted elution time
Fitting R2: 0.846
Novel peptide residual Z score: 2.02
Number of peptides: 3162
Pred. hydrophobicity
60
40
20
0
0
20000
40000
60000
Experimental scan number
C
Distributions of residuals from best−fit line
of predicted RT vs Expt. scan number
Line: Z score of novel peptide
Z: 2.02
count
750
500
250
0
−10
−5
0
5
10
Z scores of residuals from best linear fit
